# A Review on the Biocompatibility of PMMA-Based Dental Materials for Interim Prosthetic Restorations with a Glimpse into Their Modern Manufacturing Techniques

**DOI:** 10.3390/ma13132894

**Published:** 2020-06-28

**Authors:** Silviu Mirel Pituru, Maria Greabu, Alexandra Totan, Marina Imre, Mihaela Pantea, Tudor Spinu, Ana Maria Cristina Tancu, Nicoleta Olivia Popoviciu, Iulia-Ioana Stanescu, Ecaterina Ionescu

**Affiliations:** 1 Department of Professional Organization and Medical Legislation-Malpractice, “Carol Davila” University of Medicine and Pharmacy, 020021 Bucharest, Romania; silviu.pituru@umfcd.ro; 2 Department of Biochemistry, Faculty of Dental Medicine, “Carol Davila” University of Medicine and Pharmacy, 020021 Bucharest, Romania; maria.greabu@umfcd.ro (M.G.); alexandra.totan@umfcd.ro (A.T.); 3 Department of Complete Denture, Faculty of Dental Medicine, “Carol Davila” University of Medicine and Pharmacy, 020021 Bucharest, Romania; marina.imre@umfcd.ro (M.I.); anamaria.tancu@umfcd.ro (A.M.C.T.); 4 Department of Fixed Prosthodontics and Occlusology, Faculty of Dental Medicine, “Carol Davila” University of Medicine and Pharmacy, 020021 Bucharest, Romania; 5 Department of Orthodontics and Dento-Facial Orthopedics, Faculty of Dental Medicine, “Carol Davila” University of Medicine and Pharmacy, 020021 Bucharest, Romania; olivia.popoviciu@umfcd.ro (N.O.P.); ecaterina.ionescu@umfcd.ro (E.I.); 6 Department of Physiology, Faculty of Dental Medicine, “Carol Davila” University of Medicine and Pharmacy, 020021 Bucharest, Romania; iulia.stanescu@umfcd.ro

**Keywords:** biocompatible materials, poly(methyl methacrylate), interim dental prosthesis, three-dimensional printing

## Abstract

This paper’s primary aim is to outline relevant aspects regarding the biocompatibility of PMMA (poly(methyl methacrylate))-based materials used for obtaining interim prosthetic restorations, such as the interaction with oral epithelial cells, fibroblasts or dental pulp cells, the salivary oxidative stress response, and monomer release. Additionally, the oral environment’s biochemical response to modern interim dental materials containing PMMA (obtained via subtractive or additive methods) is highlighted in this review. The studies included in this paper confirmed that PMMA-based materials interact in a complex way with the oral environment, and therefore, different concerns about the possible adverse oral effects caused by these materials were analyzed. Adjacent to these aspects, the present work describes several advantages of PMMA-based dental materials. Moreover, the paper underlines that recent scientific studies ascertain that the modern techniques used for obtaining interim prosthetic materials, milled PMMA, and 3D (three-dimensional) printed resins, have distinctive advantages compared to the conventional ones. However, considering the limited number of studies focusing on the chemical composition and biocompatibility of these modern interim prosthetic materials, especially for the 3D printed ones, more aspects regarding their interaction with the oral environment need to be further investigated.

## 1. Introduction

An interim restoration represents a fundamental phase in oral prosthetic rehabilitation, aiming to protect the prepared teeth and surrounding soft tissues before a permanent restoration placement and helping the patient to perform essential functions throughout the period amid teeth preparation and application of the final restoration [1,2,3,4]. Interim prosthetic restorations are obtained from different types of dental materials, including conventional ones (based on monomethacrylates or acrylic resins and dimethacrylates or bis-acryl/composite resins), as well as the modern ones, which are obtained via a subtractive technology (milled PMMA) or via an additive technology (3D printed resins) [2,3,4]. Patients usually wear interim restorations for a short amount of time during the prosthetic treatment. However, there are situations when the use of provisional restorations is extended, such as complex oral rehabilitations, where multidisciplinary collaboration is needed (orthodontic, endodontic, periodontal, and surgical treatments), thus leading to prolonged usage of the provisional restorations [5].

Provisional prosthetic restorations allow for the testing of different functional and biological parameters: aesthetics, occlusal vertical dimension, new occlusal static and dynamic scheme, implant integration, gingival healing and re-shaping, etc. Thus, provisional restorations facilitate and support the remodeling and repair of the periodontal tissues, particularly when periodontal treatment precedes the application of the prosthetic treatment. In addition, interim restorations are used to restore functionality and aesthetics after dental extractions and until the implant insertion [6]. Provisional restorations successfully contribute to the guidance of the healing process of peri-implant gingival tissue, which facilitates an optimal emergence profile. It is mandatory to choose a biocompatible material to fabricate a provisional restoration that has no negative/adverse effects on the periodontal tissue.

To this extent, as sometimes the prosthetic treatment requires specific planning or some unpredictable situations may occur (illness, quarantine), provisional restorations have to remain in situ for a long period. Apart from their mechanical properties, provisional restorations should exhibit minimal invasive interaction with the oral environment; thus, their chemical composition and interaction with the oral environment need continuous evaluation.

PMMA (poly(methyl methacrylate)), the most commonly used conventional acrylic provisional, is also included in the formula of modern provisional materials (milled PMMA and 3D printed materials). Conventional interim prosthetic restorations are usually manufactured using resin-based materials: methacrylate resins (liquid/powder, hand-mixed) or composite resin-based materials (paste/paste, mainly auto-mixed) [7].

Methacrylate resins have a chemically initiating reaction (self-curing), while composite-based materials can be found either as self-curing or dual-curing systems [8]. Self-curing PMMA is well known for direct provisional restorations. Low costs, aesthetic qualities, good wear resistance, high polishability, color stability, a good marginal fit with optimal transverse strength, and biocompatibility with the oral tissues confirm PMMA as being a highly suitable material for direct interim restorations and making it one of the most used materials for the manufacturing of durable provisionals [9]. On the other hand, the new manufacturing techniques (subtractive and additive technologies) used for obtaining indirect interim prosthetic restorations have already proven their valuable biomechanical properties.

Nevertheless, PMMA-based dental materials exhibit several disadvantages that have to be taken into consideration. For instance, there is scientific evidence showing that self-curing PMMA monomers can have a deleterious effect on the oral mucosa and the skin [10], especially when direct techniques for obtaining interim prosthetic restorations are used; its contraction and exothermic reaction during polymerization could also interfere with the oral status. Moreover, in certain circumstances, such as parafunctions or specific anatomic structures, the mechanical behavior of PMMA-based materials is not impressive, which may cause fissures and fractures to occur. Furthermore, given the biochemical structure lacks ionic charge and favors hydrophobic and electrostatic interactions, PMMA-based dental materials promote bacterial contamination. In addition, PMMA is radio-transparent, susceptible to deterioration when in contact with water, and has low thermal conductivity, thus influencing taste perception [11].

In this context, the main objective of this paper is to synthesize the basic aspects, both favorable and unfavorable, that are related to the chemical composition, oral biocompatibility, and cytotoxicity of PMMA-based dental materials for interim prosthetic restorations, as well as to additionally point out modern interim prosthetic materials’ qualities and the potential for further development.

## 2. PMMA Chemistry

**Figure 1:**
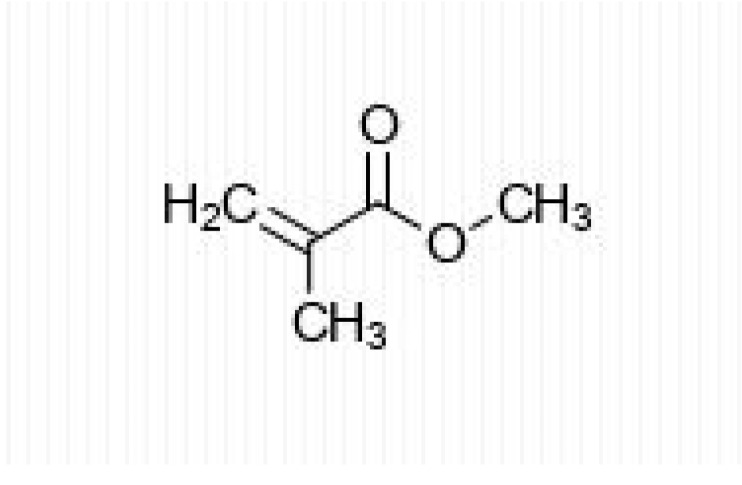
Methyl methacrylate (MMA) structure.

Methacrylic acid (MAA) and the lower alkyl methacrylates are chemical compounds produced on a large scale [12]. Methyl methacrylate (MMA) (Figure 1) is the methyl ester of MAA (methyl 2-methylpropenoate) and is a colorless liquid. MMA is the highest-volume compound among lower methacrylates used for polymer synthesis. Therefore, there is a risk associated with human exposure, leading to serious concerns about the negative effects it has on health.

**Figure 2:**
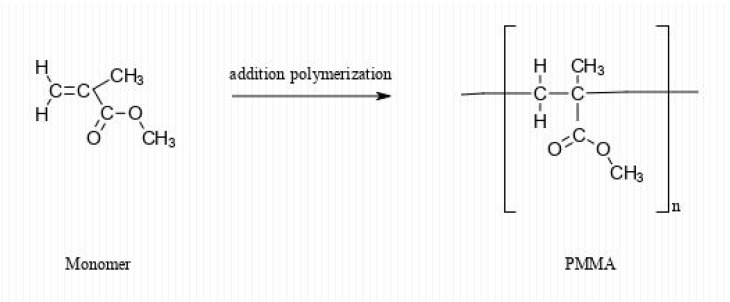
MMA polymerization is used to obtain poly(methyl methacrylate) (PMMA).

MMA is a monomer produced on a large scale for the formulation of PMMA (Figure 2).

PMMA was discovered in the early 1930s by British chemists Rowland Hill and John Crawford at Imperial Chemical Industries [13]. Methacrylates in methacrylate-based dental materials are stable polymers that do not decompose in constituent monomers. However, the non-polymerized monomers may accompany the polymer molecule, exposing humans via contact or inhalation (if the monomers are volatile) [13].

Unlike acrylic acid (AA), MAA and its alkyl esters have a methyl group adjacent to the double bond in the acyl component, hindering nucleophile addition at this site [14]. This nucleophile addition is correlated with decreased cytotoxicity and genotoxicity of methacrylates compared to the acrylates [15,16,17].

## 3. PMMA-Based Dental Materials’ Interaction with Oral Cells and Tissues

The complex interactions between provisional prosthetic materials and soft oral tissues, namely, the gingival epithelium and oral mucosa, represent extremely important aspects in maintaining a favorable equilibrium of the oral environment. The health of the gingiva located near a provisional crown is influenced by several factors, among which, the material’s characteristics/properties are of utmost importance [5,18,19,20].

Cells and tissues in contact with a biomaterial will respond in a way that depends on the biomaterial surface properties [21,22]. The biomaterial surface topography determines the cellular response and has to be regarded as a key element in the development of biomaterials [23,24,25,26]. However, the mechanisms through which surface chemical properties induce cellular responses remain to be clarified.

Oral epithelial cells and fibroblasts usually indirectly interact with biomaterials through extracellular matrix (ECM) proteins adsorbed onto their surface [27]. Consequently, it can be hypothesized that the biomaterial’s surface proteomic signature might decide the nature of its interaction with cells.

A study by Abdallah et al. revealed that biomaterials with surface chemical properties related to that of percutaneous tissues, such as aminated PMMA, induced higher selective adsorption of key basal lamina proteins (laminins, nidogen-1), subsequently improving their interactions with the epithelial cells. These experimental findings suggest that imitating the surface chemistry of epithelial cells could be a way toward improving the biomaterial–epithelial interaction [27].

It is noteworthy that most biomaterials’ failure originates from the inability to control their surface’s influence on cellular behavior, subsequently triggering unfavorable host responses [27]. In other words, the specific way a biomaterial selectively interacts with specific ECM proteins of a given tissue decides the nature of the cell–biomaterial interactions [27].

Biomaterial exposure to tissues leads to the rapid adsorption of ECM proteins onto the biomaterial’s surface [28,29]. Biomaterial surface properties determine the composition, conformation, and amount of the initially adsorbed proteins. Moreover, the surface properties also control the process of secondary protein exchange and cell-mediated surface-protein turnover [21,28]. However, a real biomaterial–epithelial integration has not been observed yet, probably due to the incomplete understanding of epithelial–biomaterial interactions.

Naturally, epithelial cells adhere to the surface of percutaneous tissues (such as nails and teeth), building a seal that can prevent bacterial invasion [30,31,32]. This seal, named the junctional epithelium, is formed during organogenesis but it can be re-activated following treatment for infections or trauma treatments [33]. Normally, oral epithelial cells–teeth interactions occur via an ECM protein complex, known as a basal lamina. This protein complex is quite rich in adhesive proteins, like laminins [27,34]. Given that basal lamina proteins are very important for epithelial cell adhesion onto the teeth surfaces, where these proteins should also be outlined as crucial for the epithelial–biomaterial interaction.

In their study conducted on epithelial cells incubated with flat-surfaced samples of PMMA, Abdallah et al. have shown that greater amounts of basal lamina proteins were adsorbed onto PMMA and alginate compared to other biomaterials. It has also been shown that in the case of gingival epithelial cells, PMMA promoted higher cell viability on day 1 than all other biomaterials studied. Moreover, on days 3 and 7 of incubation, PMMA, along with PDLLA (poly(D,L-lactic acid)) and alginate, induced higher epithelial proliferation rates compared to other biomaterials [27]. In addition, Abdallah et al.’s results also highlighted that preparing the surface chemistry of a biomaterial closer to that of the contact tissues (i.e., PMMA amination) enhanced the key basal lamina proteins (keratin 5; laminin-a, b, c; and nidogen-1) adsorption. Consequently, this surface preparation had a positive effect on epithelial cell attachment and proliferation [27].

Laminins represent the major link between basal epithelial cells and the underlying structures and are the main actors in the gingival epithelium wound-healing stage [35,36]. Among laminins, laminin 332 is distinctive because it is required to initiate hemidesmosomes’ formation and to re-establish epithelial anchorage [35,37]. This is the reason why laminin 332 coatings increase epithelial attachment onto biomaterials [38].

Keratins are intermediate filament proteins that are preferentially expressed by epithelial cells. They are important for maintaining epithelial cells’ integrity and viability. More importantly, keratins are key players for cell-matrix adhesion and migration [39,40]. Considering that the basal epithelial cells interact with keratins (keratin 5 and keratin 14) through the hemidesmosomes and the hemidesmosomal attachments are essential in the junctional epithelium, this explains why biomaterials with a higher affinity for keratin 5 induce a better epithelial attachment [27,39].

Abdallah et al.’s study revealed that regarding gingival fibroblasts, on day one of incubation, PMMA also induced a higher percentage of cell viability compared to other biomaterials. However, PMMA-incubated samples revealed a reduced number of viable gingival fibroblasts on day 7. The seventh-day fibroblast proliferation rate was negatively correlated with the contact angle measurement and positively correlated with the surface roughness [27].

A study by Mokkapati et al. highlighted that nidogen-1 was the only peptide that significantly correlated with the viability of both fibroblasts and epithelial cells. Even if working basement membranes can be generated in the absence of nidogens, the nidogen deposition removes the previous adsorption of other key proteins, like laminins [41]. Therefore, nidogen-1 can be regarded as an indicator of an optimal basal lamina deposition.

Biomaterial surface chemistry is a key player in healing around dental appliances. High-resolution X-ray photoelectron spectroscopy (XPS) analysis revealed that teeth surfaces contain nitrogen [42,43]. Because PMMA molecules do not present nitrogen in their chemical structure, it can be assumed that surface amination with diazonium compounds should improve gingival epithelial cell interactions with this biomaterial [27,44]. Abdallah et al. confirmed that amination improved PMMA surface’s chemical properties in order to become more beneficial for the teeth, favoring the interactions with both gingival epithelial cells and fibroblasts [27].

It is generally accepted that an ideal biomaterial surface should induce epithelial interactions to build the first antibacterial barrier and enhance the interactions with fibroblasts to generate a connective tissue matrix, which is necessary for supporting the overlying epithelial layer and preventing its down-growth. Abdallah et al.’s experimental results led to the conclusion that PMMA may be regarded as a promising candidate for non-degradable dental devices. However, future research work is needed to obtain the optimal surface chemical properties such that they can sustain favorable interactions of this biomaterial with oral cells [27].

## 4. Possible Toxic Effects of PMMA-Based Dental Materials on Oral Cells

Conventional acrylic resin is the most used material for the manufacture of provisional restorations due to its affordable cost, easier manipulation, good mechanical properties, and esthetic outcomes. However, it has elevated polymerization shrinkage [3,5], residual monomer release, and high stainability [3]. The acrylic-based self-polymerizing resin is obtained from a pre-polymerized PMMA powder and liquid MMA monomers [1].

In vitro studies revealed that MMA monomer-to-polymer conversion is incomplete. Consequently, unpolymerized monomers can be released into the oral cavity, where they will interact with the oral cells [45]. Moreover, clinical studies conducted on patient saliva samples collected after dental restorative procedures have highlighted MMA monomers’ release after polymerization [46,47,48,49,50]. Some studies have illustrated the PMMA-based materials’ adverse effects on oral tissue and at a cellular level [51]. PMMA-based dental materials’ adverse effects on oral tissue include inflammatory reactions, fibrosis, necrosis around the materials, and pulpal responses [52,53].

Prosthetic PMMA resin interim restorations obtained via self-polymerization directly onto the prepared vital teeth could generate a biological response from the dental pulp cells. In the case of deep cavities, the small and hydrophilic unpolymerized MMA monomers, along with other low-weight compounds released during polymerization can diffuse across the remaining dentin layer and induce adaptive pulp cell responses [1,54,55].

Studies conducted on primary cultured dental pulp, gingiva, and periodontal ligament cells have highlighted that at the cellular level, both the polymerized PMMA resin and the unpolymerized MMA monomers induced genotoxic effects, cell cycle disturbances, and even apoptosis. Moreover, it has also been noticed that the presence of both PMMA resin and MMA monomers altered the normal mineralization processes [56,57,58]. It can be concluded that the molecular mechanisms of these toxic effects have the residual MMA monomers release as a starting point, which will initiate free radicals’ formation, and finally, the generation of oxidative stress [56,59].

Oxidative stress represents the imbalance between reactive oxygen species (ROS) production and their inactivation by the antioxidant protective mechanisms [60]. Oxidative stress induces the activation of a variety of transcription factors, which lead to the differential expression of specific genes involved in inflammatory pathways. All these events lead to inflammatory reactions [61]. In return, inflammation itself will amplify oxidative stress through the increased production of ROS and free radicals. Proinflammatory signals trigger the release and activation of peroxiredoxin, a ubiquitous redox-active intracellular enzyme. After its release, this enzyme will play the role of a redox-dependent inflammation mediator and will activate tumor necrosis factor-α (TNF-α) synthesis and release from macrophages [61]. Before or during release, the peroxiredoxin molecules are glutathionylated in the presence of reduced glutathione (GSH). It has been shown that this process is an important player in the immunity regulation stage [62]. It has also been noted that macrophage peroxiredoxin, along with thioredoxin, can disturb the redox status of cell surface receptors, and consequently, induce an inflammatory reaction [61].

Moreover, studies have shown that cells’ exposure to MMA monomers enhanced ROS generation, and simultaneously, decreased the intracellular GSH [56]. These findings show that exposure to MMA monomers could be a starting point in oxidative stress initiation. At high levels, ROS can overwhelm cellular antioxidants, opening the way to oxidative stress and initiate oxidative attacks on important cellular macromolecules, such as proteins, lipids, and DNA [63].

Temporary intracellular ROS accumulation represents the first major cellular response to environmental aggression, including MMA monomers’ presence. Moreover, high levels of ROS were also detected after a short exposure of cell cultures to the PMMA resin, probably due to the remaining unpolymerized MMA monomers [1,64]. These experimental results could represent the first steps toward the explanation of PMMA resins’ adverse effects on oral tissues.

Studies conducted on oral cells exposed to MMA revealed significant alterations in the activities of antioxidative enzymes, such as catalase, superoxide dismutase, and glutathione peroxidase. Increased levels of a lipid peroxidation product, namely, malondialdehyde, have also been reported [1,64]. It is quite important to highlight that these antioxidant enzymes’ gene expressions are controlled by erythroid 2–related factor 2 (Nrf2) [65]. Nrf2 represents a key regulator of cellular resistance to oxidative attacks. This regulator coordinates the basal and induced expression of genes depending on an array of antioxidant response elements, which are involved in the elaboration of physiological and pathophysiological responses to oxidative stress. Studies have shown that the activation of a Nrf2-regulated cellular antioxidant defense overwhelms the resin-monomer-induced oxidative stress and ensures cell viability [66,67]. Furthermore, Zhang’s studies showed that the pharmacological activation of Nrf2 by tertiary butylhydroquinone protected the PMMA-resin-exposed cells from apoptosis, illustrating a possible new alternative therapeutic target for maintaining oral cell functions under MMA-induced oxidative stress [1].

One of the main consequences of oxidative stress is DNA damage. Szczepanska et al. have shown that in vitro exposure of human gingival fibroblasts to MAA monomers leads to DNA damage after 6 h of incubation [68]. At DNA damage sites, the histone H2AX phosphorylation to γ-H2AX represents an early step in the elaboration of the cellular response to DNA double-strand breaks (DSBs). Experimental data reveal that H2AX phosphorylation is an indicator of DNA DSB formation in cells exposed to an MMA monomer resin [69,70]. Normally, cells respond to DNA damage by blocking the cell cycle and activating cell cycle checkpoints to initiate the DNA repair process. Zhang et al. have shown that MMA exposure triggered a corresponding delay of the cell cycle in the G1 phase [1]. Their observation also highlights that the G1 phase checkpoints are very sensitive to oxidation-induced DNA damage [1,71]. Furthermore, the ataxia telangiectasia mutated (ATM) protein can be recruited and activated by DNA DSBs, triggering tumor protein P53 (P53) activation [72]. Protein P53, the genome guardian and a crucial tumor repressor, is a key player in cell cycle control, DNA repair processes, and apoptosis initiation [73].

In response to oxidative stress, P53 acts as a transcriptional activator to promote the expression of cyclin-dependent kinase inhibitor 1 (P21). Activated P21 acts as an inhibitor of cell cycle progression at the G1 phase [74]. Zhang et al. illustrated that ATM, P21, and P53 gene expressions were significantly increased in cell cultures exposed to the PMMA resin [1]. The ATM-activated P53 also controls the transcription of pro- and anti-apoptotic proteins. In this regard, Zhang has shown that in pulp cells exposed to PMMA for 24 h, the B-cell lymphoma 2 (Bcl-2) family of proteins changed their usual localization. PMMA resin exposure also triggered mitochondrial dysfunction, cytochrome C release, caspase machinery activation, and consequently, cell death via apoptosis [1,64,75].

Moreover, P53 can control phosphatase and tensin homolog (PTEN), which in turn is a negative regulator of the phosphatidylinositol 3-kinase (PI3K) pathway that translates signals from tyrosine kinase receptors for cellular event modulation. Normally, the activated PI3K phosphorylates protein kinase B (AKT) and stimulates the mammalian target of rapamycin (mTOR) pathways [76]. mTOR activation favors the phosphorylation of certain proteins that are responsible for cell proliferation [77]. P53 activation via ATM signaling, which is a consequence of oxidative DNA damage, triggers the Akt/mTOR pathway downregulation through PTEN activation. Consequently, cell proliferation is blocked and the apoptotic events are initiated.

All these experimental data led us to the conclusion that self-polymerized PMMA resins, despite their advantages, can induce oxidative stress in the exposed pulp cells (most probably due to the remaining unpolymerized MMA monomers), which causes oxidative DNA damage, which in turn is a starting point for apoptotic events triggering via P53 and PTEN signaling pathways [1,78].

As a preliminary conclusion, the collected literature data indicate that PMMA-based materials used for interim prosthetic restorations raise certain problems concerning their characteristics and their interaction with the oral environment. However, their basic valuable properties are widely recognized. For instance, conventional PMMA-based materials have good esthetics, low cost, easy manipulation and repairing, and clinically accepted oral biocompatibility.

On the other hand, the development of digital dentistry has led to remarkable progress in the last few years, creating new opportunities in the restorative dentistry [10,79,80]. In particular, subtractive technology and additive manufacturing (AM) technology represent significant achievements in the field of digital dentistry, offering numerous advantages compared to the conventional manufacturing methods, such as low material waste and easy mass customization. A computer-aided design/computer-aided manufacturing (CAD/CAM) system can reduce the specific shortcomings mentioned above and accelerate the manufacturing process of prosthetic restorations. Recent scientific studies have also begun to focus on improving the use of 3D printing in obtaining successful interim prosthetic restoration [11].

A limited number of in vitro studies have focused on evaluating the provisional materials’ biocompatibility [18,59]. According to Campaner et al., the most negative effects were observed on gingival fibroblasts in mice and were induced by auto-polymerized acrylic resin and bis-acrylic resin, while the CAD/CAM nano-ceramic resin and prefabricated polymer block caused the production of the smallest amount of interleukin-6 (IL-6), interleukin-1β (IL-1β), and TNF-α, therefore proving to be the most appropriate provisional materials for preserving the health of the tissues surrounding the teeth [81]. Shim et al. [82] recommend CAD/CAM technology and indirect fabrication of interim prosthetic restorations supported on implants to prevent residual monomer release and to achieve high cell attachment. Additionally, Engler et al. [83] found that the level of residual monomer elution for milled PMMA used for obtaining interim prosthetic restoration was below the one corresponding to conventional polymers. Along the same line, Atay et al. [84] pointed out that new generations of CAD/CAM provisional restoration materials (milled PMMA) can be safely used in clinical conditions and Herráez-Galindo et al. [85] suggested that milled PMMA demonstrated a fibroblastic behavior similar to those of lithium disilicate, which was considered the “gold standard”.

Furthermore, the use of AM has been considered a viable alternative for biomedical implants and tissue engineering for treating both systemic diseases, as well as oral disorders [86,87]. A digital dental prosthetic workflow includes 3D printing to manufacture clinically accurate provisional restorations. The American Section of the International Association for Testing Materials (ASTM) states the technical requirements for various types of materials and products and has established seven categories of AM methods: stereolithography (SLA), material jetting (MJ), material extrusion or fused deposition modeling (FDM), binder jetting, powder bed fusion, sheet lamination, and direct energy deposition. These 3D printing methods differ from each other in terms of the final product characteristics (geometric accuracy, surface finishing, design, structure, mechanical behavior), selection of proper material, layer bonding methods, or production efficiency [88].

However, the potential of 3D printing is challenged by the difficulty in finding proper formulations of the biomaterials that ensure biocompatibility and optimal mechanical properties [86,87]. Regardless of the fairly considerable number of recent review papers debating the use of 3D printing in dentistry [89], there is an outstandingly low amount of data corresponding to the properties and characteristics of 3D-printed restorative dental materials [90,91].

A reduced number of AM polymers recommended for provisional prosthetic restorations are officially available for intraoral use; these materials usually comprise monomers based on acrylic esters or filled hybrid material. Provisional AM materials abide by the same classification as conventional interim materials, namely, mono methacrylates or acrylic resins and dimethacrylates or bis-acryl/composite resins, such as bisphenol A-glycidyl dimethacrylate and urethane dimethacrylate (these resins are polymerized by light). Nonetheless, part of the chemical composition of the AM provisional monomers has not been disclosed by manufacturers [92]. As specified in the available studied literature on this matter, one of the 3D-printed materials approved for interim dental restorations, namely, Temporis/DWS, contains a mixture of multi-functional acrylic monomers and esters of acrylic acid. The chemical composition of E-Dent100/Envisiontec is represented by tetrahydrofurfuryl methacrylate, urethane dimethacrylate, phosphinoxide, and multifunctional acrylic resins; E-Dent400 includes a monomer based on acrylic esters. Stratasys produces VeroGlazeMED620, which contains 2-hydroxy-3-phenoxypropyl acrylate, 4-(1-oxo-2-propenyl) morpholine, exo-1,7,7-trimethylbicyclo[2.2.1]hept-2-yl acrylate, tricyclodecane dimethanol diacrylate, bisphenol-A epoxy acrylate oligomer and (2,4,6- trimethylbenzoyl)diphenylphosphine oxide. The chemical compositions of other 3D-printed interim materials approved for clinical use, such as Freeprint/Detax, C&B, or C&B MFH/Nextdent, are not yet revealed by the producers. Additionally, the inorganic filler is described only for E-Dent 100/Envisiontec: 49.8 (0.04–0.7 μm particle size of inorganic fillers) [92].

Along with a proper chemical composition that assures good oral biocompatibility, different elements are important in obtaining predictable, successful, accurate interim 3D prosthetic restorations, as follows [10,90,92,93,94,95]: the number of printed layers; the shrinkage that appears between the printed layers; laser speed, intensity, angle, and building direction; type of used software; the position and angle of the restoration on the printing platform; the amount of supportive material; and post-processing procedures. In this context, it is widely recognized that discrepancies regarding the printing parameters and technical printing protocols have been noticed, and as the chemical compositions of the 3D printed materials are not yet completely provided by the manufacturers, comparing the different available studies’ results could be considered particularly challenging.

As the list of CAD/CAM or printable interim dental materials expands, the rapid growth of digital technologies will certainly continue. While there is an increasing demand for all of these high-tech restorations, extensive clarifying details are needed regarding the chemical composition and mechanical properties of the above-mentioned new materials [11]. Future studies on the modern systems used for obtaining interim prosthetic restorations should be developed to optimize the clinical use, as well as to enhance the technical parameters, especially for AM. Therefore, prosthodontists could personalize the selection of dental materials for interim prosthetic restorations corresponding to each specific clinical case.

## 5. Conclusions and Future Perspectives

PMMA, which is usually included in the composition of conventional materials used for interim prosthetic restorations, as well as in the modern ones obtained using subtractive or additive technologies, has already proved its clinical performance in the context of obtaining predictable, final prosthetic results, even if their interaction with the oral environment has always been a subject of great interest and debate in the scientific world.

Significant aspects concerning the oral biocompatibility of PMMA-based dental materials used for obtaining interim prosthetic restorations were presented in this paper. Elements regarding adverse effects potentially caused by PMMA-based dental materials on epithelial cells, fibroblasts, or pulp cells, along with their influence on the oxidative stress response and residual monomer release, were synthesized to collate their acknowledged, recognized qualities. Recent encouraging data collected from studies focusing on modern subtractive and additive techniques used to obtain interim prosthetic restorations were also presented.

Comprehending how well conventional, milled, or printed materials for interim prosthetic restorations relate to the oral environment allows dental professionals to develop more effective, successful treatment planning, further increasing the quality of oral care. Modern manufacturing technologies show a promising future in prosthetic dentistry based on their remarkable development potential; therefore, further in vitro and in vivo studies are needed to sustain the fast implementation of these materials in clinical dentistry.
